# Digital LED Pixels: Instructions for use and a characterization of their properties

**DOI:** 10.3758/s13428-015-0653-5

**Published:** 2015-10-20

**Authors:** Pete R. Jones, Sara E. Garcia, Marko Nardini

**Affiliations:** 1 Institute of Ophthalmology, University College London (UCL), 11-43 Bath Street, London, EC1V 9EL UK; 2 Department of Psychology, Durham University, Durham, UK

**Keywords:** Light emitting diode, Arduino, Luminance, Timing, Color gamut

## Abstract

This article details how to control light emitting diodes (LEDs) using an ordinary desktop computer. By combining digitally addressable LEDs with an off-the-shelf microcontroller (Arduino), multiple LEDs can be controlled independently and with a high degree of temporal, chromatic, and luminance precision. The proposed solution is safe (can be powered by a 5-V battery), tested (has been used in published research), inexpensive (∼ $60 + $2 per LED), highly interoperable (can be controlled by any type of computer/operating system via a USB or Bluetooth connection), requires no prior knowledge of electrical engineering (components simply require plugging together), and uses widely available components for which established help forums already exist. Matlab code is provided, including a ‘minimal working example’ of use suitable for use by beginners. Properties of the recommended LEDs are also characterized, including their response time, luminance profile, and color gamut. Based on these, it is shown that the LEDs are highly stable in terms of both luminance and chromaticity, and do not suffer from issues of warm-up, chromatic shift, and slow response times associated with traditional CRT and LCD monitor technology.

**Electronic supplementary material:**

The online version of this article (doi:10.3758/s13428-015-0653-5) contains supplementary material, which is available to authorized users.

## Introduction

To present visual stimuli, psychophysicists in the 19^th^ and 20^th^ century developed many ingenious methods, including the use of spinning tops (Maxwell 1857), shadow-casting by lamps or candles (Mach 1959; Fry 1948), and mechanical systems in which viewable objects are physically translated in space (Tschermak-Seysenegg 1939; Howard 2012).

**Fig. 1:**
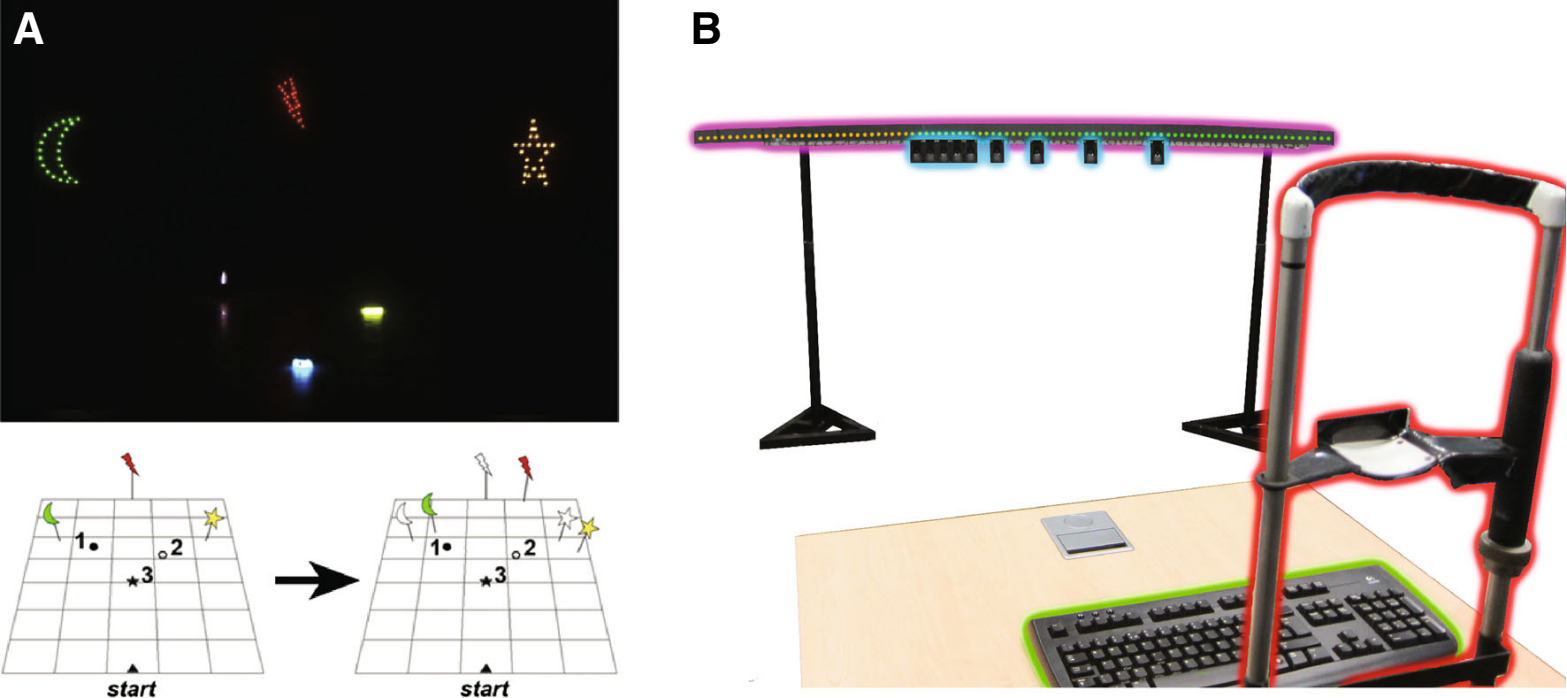
Two example uses of LEDs in behavioral experiments. **a** Illuminated visual landmarks for a study of navigation (image adapted from Nardini et al. (2008)). Duplicate sets of landmarks (shown lower-right of panel a) were positioned at regular intervals around the room, and could be switched on/off, effectively rotating the landmarks with respect to the participant. The objects on the floor were illuminated using chemiluminescent paint. Now, though, these movable objected could be instead fitted with LEDs and wireless microcontrollers. **b** A 2.5-m-long arc of lights and speakers, for a study of audiovisual localization (used in Garcia et al. (2015)). On each presentation, a subset of the LEDs (*outlined in purple*) and/or speakers (*outlined in blue*) at a particular location were activated. The LED hardware used was identical to that described in the present paper, and allowed each of 100 LED lights to be controlled independently using commands sent from a central control computer (not shown)

Modern-day scientists typically prefer to use computer monitors to present their visual stimuli. Computer monitors are particularly effective at presenting high-resolution static images in the central field. However, they are less well suited to other applications; for example, when stimuli must span the entire visual field, are physically located in an interactive 3D environment, or when high temporal precision is required. In these cases, light emitting diodes (LEDs) can provide a surprisingly simple and effective solution (Nygaard and Frumkes 1982; Da Silva Pinto et al. 2011; Teikari et al. 2012; Demontis et al. 2005; Albeanu et al. 2008). For example, in our own research, we have found LEDs useful for constructing dynamically adjustable landmarks for studying human navigation (Fig. 1a), and for presenting peripheral stimuli in an audiovisual localization task (Fig. 1b).

In the past, many researchers have been discouraged from using LEDs because of the level of electrical engineering required. Wires must be soldered together, the level of electrical current must be regulated appropriately, and control circuits must be designed and constructed to produce the specific behavior required. Furthermore, since the equipment is typically made bespoke for each specific experiment, it is typically expensive, inflexible, and hard to maintain.

Recently though, these difficulties have been obviated by the proliferation of cheap LED ‘Pixels’. Unlike traditional LEDs, an LED Pixel integrates all the necessary electronic components into a single, prefabricated device. This means that they simply need to be plugged in for use. Furthermore, each LED Pixel contains its own digital control chip that can receive instructions from a computer. The LED Pixel can therefore be controlled purely at a software level, in much the same way as a psychophysicist might control a standard computer monitor (e.g., using commands sent from Matlab or Python) Multiple LED Pixels can be chained together, often by simply plugging the output lead of one LED Pixel into the input lead of another. This allows effectively limitless numbers of Pixels to be controlled simultaneously. Crucially though, since every LED Pixel contains its own digitally addressable control chip, the behavior (timing, intensity) of each Pixel can be controlled independently. Finally, more advanced LED Pixels house multiple LED elements—each with a different spectral response curve—within a single, light-diffusing enclosure. By additively mixing multiple color channels, a wide range of colors and luminance levels can be produced. The result is a cheap, flexible, and easy-to-use system in which all the necessary hardware can be purchased in ready-made ‘modules’, and can be controlled programmatically, using simple, user-friendly commands.

Three hurdles have limited the uptake of this new technology within the behavioral sciences. Firstly, many researchers are unaware of these recent developments. Secondly, the sheer number of competing brands and hardware, together with the few technical bottlenecks that remain (e.g., how to install the necessary software, or communicate reliably with the hardware), mean that exploiting such advances remains a daunting proposition for the many researchers who lack the time or expertise to sift through the range of options available. Third, and finally, there is a general uncertainty as to how the stimuli produced by LEDs relate to those generated by computer monitors, which are felt (though often erroneously^1^) to be well understood.

The present paper aims to address each of these points. In section “Methods”, we detail the necessary hardware required in order to build an operational LED Pixel system, and provide step-by-step instructions on how to assemble it. In section “Usage (with minimal working example)”, we describe the logic of how this equipment can be controlled using software, and provide a minimal working example (MWE) of use. Finally, in section “Characterization” we characterize the properties of our recommended LEDs, and show how their luminance, spectral, and timing properties compare to those of LCD and CRT monitors.

## Methods

### Hardware: Overview

**Fig. 2:**
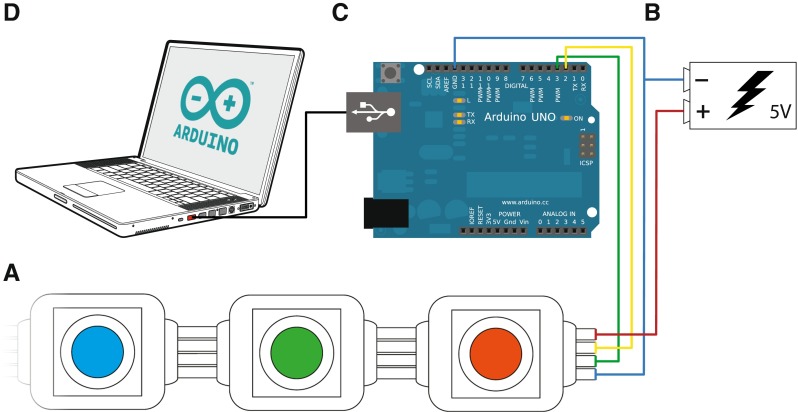
Schematic illustration of the key hardware required. **a** A strip of digitally addressable LED ‘Pixels’. Each pixel consists of three independent LEDs (*red, green, blue*), located behind a diffuser, and controlled by an internal chip (WS2801). **b** A 5-V power supply (either battery or mains adapter). **c** An Arduino Uno microcontroller, used to control the LED Pixels. **d** An ordinary laptop or PC to program, control, and supply power to the Arduino. See body text for details, and Table 1 for further particulars

The required hardware is shown graphically in Fig. 2, and is listed in Table 1 The four key pieces of equipment are the LED Pixels, a power supply, an Arduino microcontroller, and a computer. Each of these is described in more detail below.

**Table 1:** Complete listing of required hardware, including illustrations of example products

Required Hardware		Description
*N*	Image	
1		Computer
		Any latpop or desktop computer with a USB 2.0 connection (or newer).
1		Arduino Microcontroller
		A microcontroller, used to interface between the *Computer* and the
		*LED Pixel Strand* hardware. Any Arduino board is sufficient, but the
		current ‘standard’ Uno board is recommended for consistency.
1		USB 2.0 Cable
		One male (Type A) to male (Type B) USB 2.0 Cable, to transmit data between
		the *Computer* and *Arduino Microcontroller*. Also used to supply power
		to the *Arduino Microcontroller*.
1 +		LED Pixel Strand
		One or more strand of Adafruit 12mm Pixels (25 LEDs per strand).
		Multiple strands can be plugged together. First strand must be
		connected to the *Arduino Microcontroller* via *Jumper Wires*.
		To avoid power drain it is recommended that one *5V DC Power Adapter*
		is used per two strands, although fewer power supplies may be required if not
		all LEDs are illuminated at any one time.
4		Jumper Wire
		Male-to-male electrical ‘breadboard’ wires, to connect first
		*LED Pixel Strand* with the *Arduino Microcontroller* (N.B. red
		wire only required if drawing power from the board). Alternatively, any ordinary
		insulated wire can be used (but will require stripping, and stranded wires may
		require tinting to avoid fraying).
1 +		**5V Power Adapter**
		Mains transformer. Recommended one per 50 LEDs. Alternatively can use a battery
		power source, or, for a smaller number of LEDs, draw power directly from the 5V
		pin on the *Arduino Microcontroller*.
1+		Power Adapter Terminal
		Takes *5V Power Adapter* output, and connects to the built-in red
		(positive) and blue (negative) wires on the *LED Pixel Strand*.
		One required per *5V Power Adapter*.

See Fig. 1 for how these components are assembled, and for further details see the manufacturer’s guide for the Adafruit 21-mm LED Pixels

#### The LED pixels (Fig. 2a)

An LED Pixel consists of one or more LED elements, each connected to an integrated control chip. Multiple LED Pixels can be chained together, but addressed independently. Although many brands of LED Pixels exist (some of which can be used interchangeably), here we concentrate on a single product: the Adafruit 12-mm diffused LED Pixels (Adafruit Industries, New York, USA). These were preferred as they support full 24-bit color, have a reasonably high modulation rate (2.5 kHz), are well supported with an efficient software library and clear instructions, require no technical assembly (e.g., no soldering), and have proven to be reliable and robust. However, the same basic methods can be easily adapted to work with other similar products.

By default, a strand of Adafruit 12-mm diffused LED Pixels contains 25 independently addressable pixels. This number can be increased by plugging together multiple strands, or reduced if required by simply cutting off unwanted pixels using scissors. Each pixel is composed of three independent LED elements (red, green blue) housed within a circular diffuser screen (8 mm in diameter), and controlled by a 24-bit (8-bit per LED) programmable driver chip (WS2801; Worldsemi Technology, Shenzhen, China). Thus, each pixel can independently display 16.78 million (i.e., 256 x 256 x 256) possible color combinations (see section “Characterization” for full empirical characterization). The chipset that drives each pixel uses 2.5-KHz pulse width modulation (PWM) to vary luminance (i.e., luminance is controlled by rapidly flickering the light on/off, ideally at a rate beyond that which can be perceived by the human eye). For further discussion of issues relating to PWM luminance-modulation, and for users who may require a continuous light source of variable luminance, see the Supplemental Material (Section S1).

#### The 5-V power supply (Fig. 2b)

The LED Pixels require a 5-V (± 10 %) input, and each LED Pixel draws up to 60 mA at maximum luminance. The input can be constituted from four 1.2-V batteries, or a 5-V mains adaptor. A mains adaptor is generally preferred for consistency and ease of maintenance. Inputs greater than 5.5 V should not be used, and may permanently damage the LED Pixels. For long strands requiring a large current, multiple 5-V power supplies can be connected at regular intervals to limit power drain (see section “Drain and halation”). If only a small number of LED Pixels are required at any one time (e.g., two or three), and/or if high luminance is not required, then the LED Pixels can also draw their power directly from the 5-V pin on the Arduino board (i.e., connecting the red wire to the pin marked “5V” in Fig. 2).

#### The Arduino microcontroller (Fig. 2c)

A microcontroller is required to interface between the control computer and the LED Pixels (Fig. 2c). We recommend using the latest Arduino microcontroller, which at the time of writing is the Arduino Uno (SmartProjects, Strambino, Italy). The code provided here will also work with other Arduino boards, including the older Diecimila and NG variants. However, the limited memory in some other models can prove prohibitive for any but the most basic programs (e.g., for any paradigms where arrays of values must be stored on the board). Instead of an Arduino board, the same basic processes can also be implemented using more powerful devices, such as the Raspberry Pi (Raspberry Pi Foundation, Cambridge, UK), BeagleBone (Texas Instruments, Dallas, TX, USA) or PCDuino (LinkSprite Technologies, Longmont, CO, USA). Note, however, that these devices are not microcontrollers, but full application processors (miniaturized personal computers), and so can be more complicated to set up and use (e.g., requiring the installation and configuration of an operating system). Compared to the Arduino boards, they also have less established user groups and help forums, which will be a substantial limiting factor for many users.

#### The computer (Fig. 2d)

A laptop or desktop computer is required to program, and to optionally supply power to, the Arduino microcontroller (see below). Both of these functions are carried out using a single USB 2.0 cable (see Table 1). The computer is also generally used to control the Arduino during the experiment, via serial commands sent over USB. Alternatively, once programmed the Arduino can be disconnected and used autonomously (e.g., responding directly to user inputs, such as button presses, and powered using a battery cell). However, it is generally more convenient to keep the Arduino tethered to a host computer, which can then be used to process participant responses, synchronize the Arduino with other devices, and/or store experimental data. Almost any computer can be used, as long as it supports a USB 2.0 connection (see section “Software requirements” for details on operating systems).

### Software requirements

The methods described here should be compatible with all operating systems (Windows XP/Vista/7/8, Mac OS X, or Linux, all 32 or 64 bit), although users should check the official Arduino support documents if uncertain. We have tested the code given in section “Usage (with minimal working example)” on PCs running Windows XP and Windows 7, and on various MacBook Pros running OS X 10.4–10.6.

The control computer must be able to run the Arduino programming language (v1.0.6 at time of writing). This is a simplified, open-source version of C/C ++. Note that although the Arduino code is written on a computer, once compiled the code is uploaded onto the Arduino board itself for execution. Note also that the computer used to program the board does not necessarily need to be the same computer that is used subsequently to control the board via serial commands, although for simplicity we shall assume that that is the case.

In addition the control computer must be capable of sending serial commands to the Arduino over USB. In the examples given here, we use Matlab (R2012b, The MathWorks, Natick, MA, USA) to do this, via the Instrument Control Toolbox. However, the same principles can be easily adapted to work with any modern programming language (Python, proce55ing, java, c ++).

### Assembly and installation

1. Purchase the hardware. All components can be purchased from most major electronics retailers. The 12-mm diffused LED Pixels can also be purchased directly from www.adafruit.coms, which at the time of writing also sells an Arduino starter kit, containing all other necessary components. At the time of writing, the total price for all hardware (excluding the control computer), is approximately $110 (plus shipping). Each additional strand of 25 LED Pixels costs a further $50 (including additional power supplies for every 3^rd^ strand, although these may not be needed if high luminance and uniformity are not required)
2. Install the Arduino software on a computer with a USB 2.0 connection (or newer).
3. Connect the Arduino board to the computer via a USB cable
4. Configure the Arduino software appropriately, by ticking the appropriate item under “Tools => Board”, and under “Tools => Serial Port”. On some machines—most notably Macbook Pro laptops—no serial portwill be listed. In this case, it may be necessarytoinstall VirtualCom Port [VCP] drivers. At the time of writing, free versions of these drivers can be found at: http://www.ftdichip.com/Drivers/VCP.htm
5. Check that the Arduino is functioning properly by running one of the inbuilt example scripts (e.g., File => Examples => 01.Basics => Blink)
6. Install the necessary LED Pixel library (“Adafruit_WS2801”), which can be downloaded from https: //learn.adafruit.com/12mm-led-pixels/code (for details on how to install a custom Arduino library, see http://arduino.cc/en/Guide/Libraries)
7. Connect the LED Pixels to the Arduino board and power supply, using the wiring diagram shown in Fig. 2. Note that jumper cables can be used to connect to the board, while the built-in power cables on the LED Pixel strip can be screwed directly into the power adapter terminal (see Table 1). Also note that the LED Pixels are not bidirectional, and must be connected at the end marked as *input*, as per the manufacturer’s instructions.
8. Test that the LED Pixels are working by running one of the inbuilt example scripts (e.g., File => Sketchbook => libraries => Adafruit_WS2801 => strandtest.

In case of difficulties, users are advised to consult the detailed online tutorials that are available for both the Arduino microcontroller (http://arduino.cc/en/Guide/HomePage), and Adafruit LED Pixels (https://learn.adafruit.com/12mm-led-pixels/). For further assistance, the Arduino is also supported by a highly active forum dedicated to the control of LEDs (http://forum.arduino.cc/index.php?board=6.0).

## Usage (with minimal working example)

**Fig. 3:**
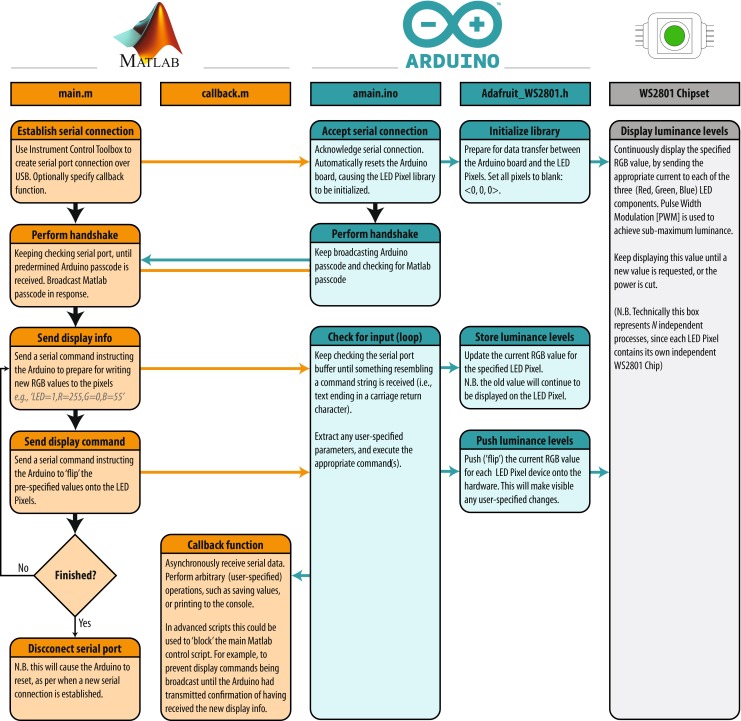
Flow chart showing how the Matlab (*orange*), Arduino (*cyan*), and LED (*gray*) systems interact. Example source code for main.m, callback.m, and amain.ino is provided in Listings 1–3, respectively. See body text for details

To be able to use the LED Pixels effectively within an experiment, it is important to be able to control their behavior precisely using a computer. This requires communication between three systems: (i) the programming environment on the control computer (here assumed to be Matlab); (ii) The Arduino microcontroller, running a preprogrammed script; and (iii) the control chip within each LED Pixel. In this section, we detail the processes necessary for achieving this. The key processes are also shown graphically in Fig. 3. Readers may also wish to refer to Listings 1, 2 and 3, which provide Matlab and Arduino code for turning a specified LED Pixel on or off. To run this code, Listing 3 must be compiled in Arduino and uploaded to the Arduino microcontroller. The code in Listings 1 and 2 must be placed in the same directory as each other. To run, Listing 1 should be executed in Matlab. The logic of this program is described in the remainder of this section.

**Listing 1:**
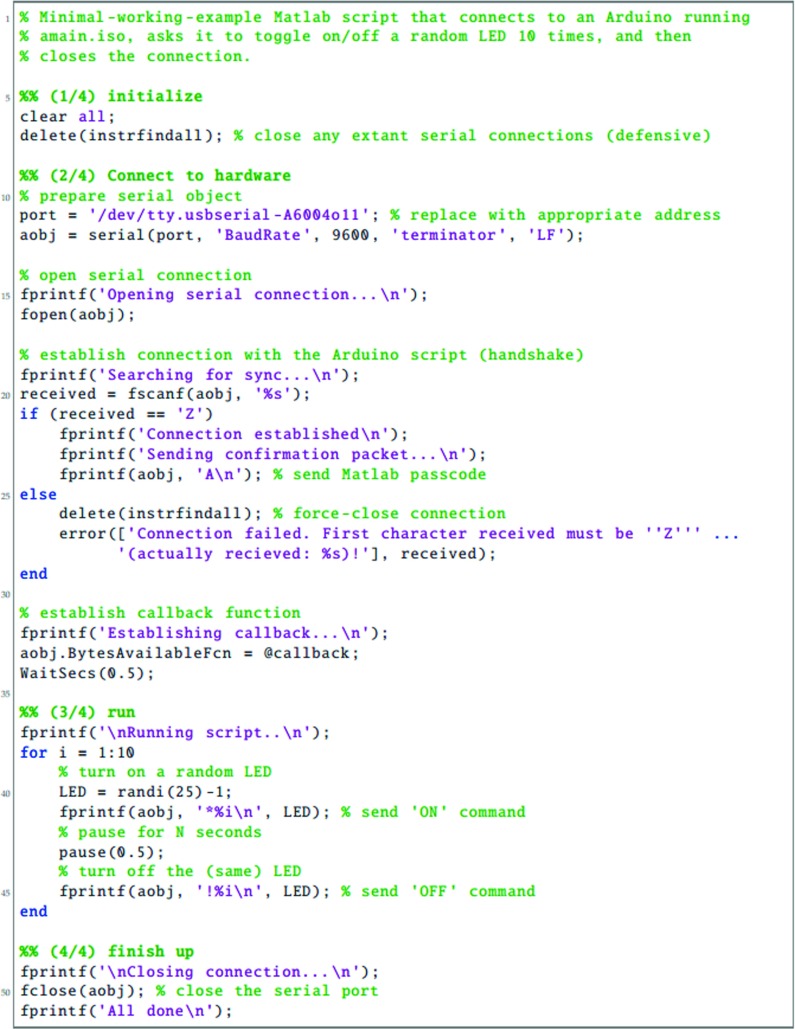
Matlab main program script (main.m), for interacting with LED Pixels via an Arduino microcontroller. See body text for details

**Listing 2:**
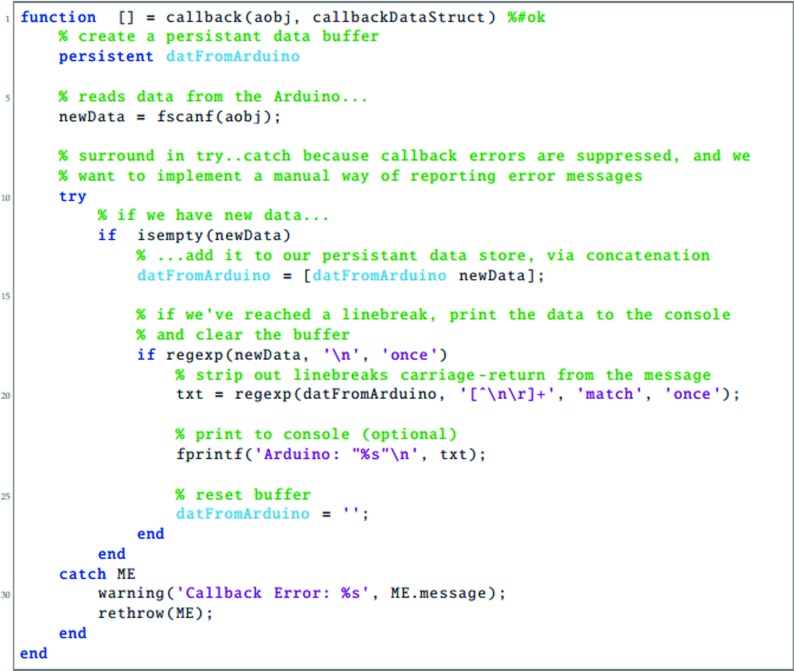
Matlab callback code (callback.m), for receiving data returned over the serial connection from Arduino. See body text for details

**Listing 3:**
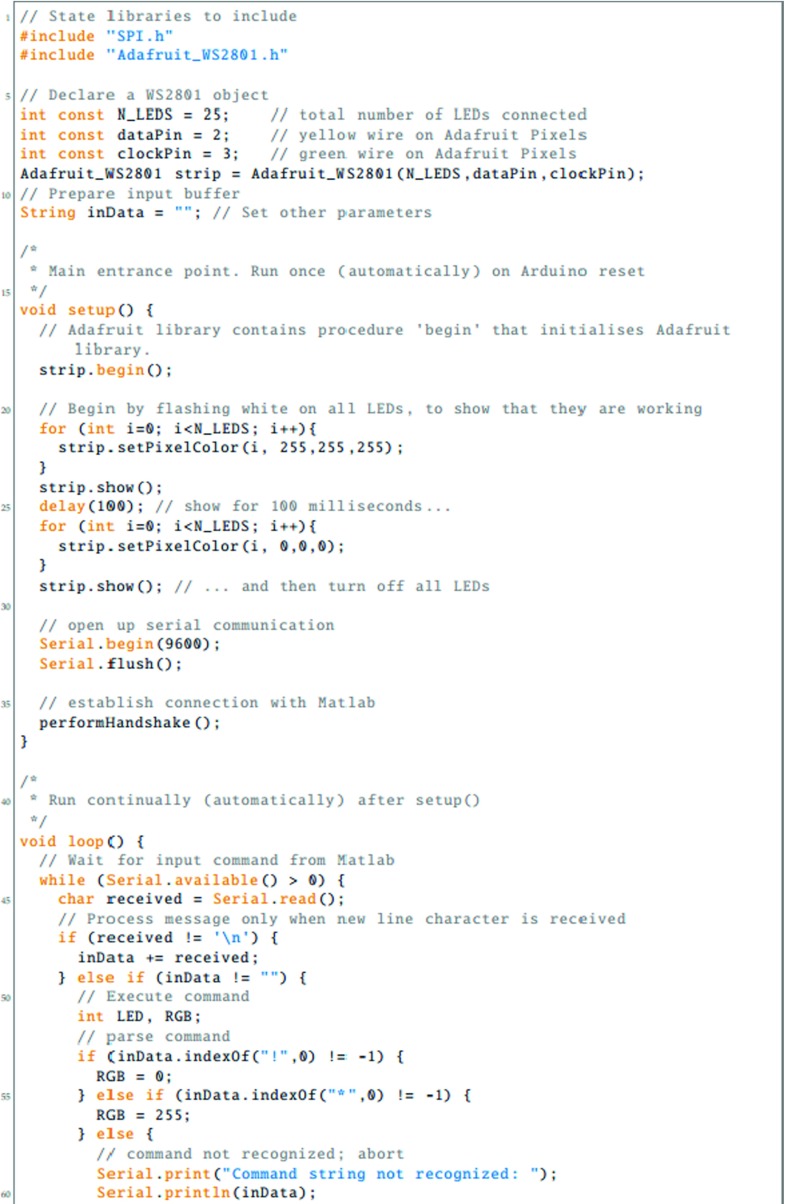

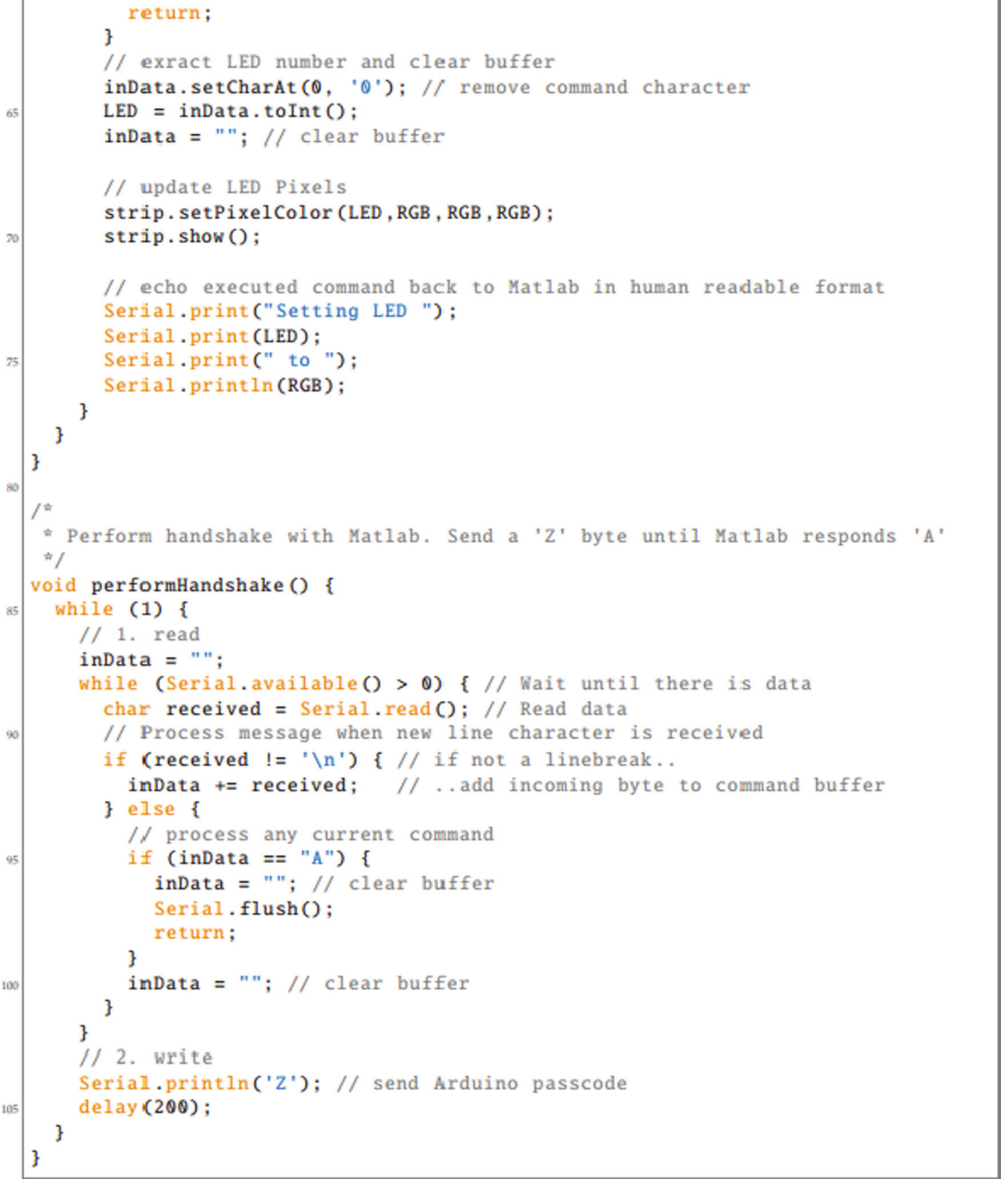
Arduino code (amain.iso), for receiving serial commands from Matlab, controlling LED Pixels accordingly, and returning status updates to Matlab

The first step is to establish a serial connection between Matlab and the Arduino board, via the USB port. This can be done most straightforwardly using the Instrument Control Toolbox(Listing 1, L10–16). The appropriate port address for the Arduino USB connection can be found by opening the Arduino IDE and clicking Tools => Serial Port. On a Apple Mac computer, the port address will generally resemble the value of ‘/dev/tty.usbserial-A6004o11’, in Windows it may resemble the string ‘COM3’. At this point, or subsequently (Listing 1, L31–34), the user can optionally specify a callback function (Listing 2). A callback function is a Matlab function that will be automatically invoked if/when the Arduino attempts to transmit serial data back to the computer. A callback function can be used to return information from the Arduino to the control computer (e.g., regarding presentation timings, or the results of any computations). Further details on the example callback function are given below.

Once the Arduino board has accepted the serial connection it will automatically reset, causing the Arduino function ‘Setup’ to run (Listing 3, L13–37). As part of this function, the LED Pixel library is initialized (with a user-specified number of pixels), causing the LED Pixels to revert to their default ‘awaiting handshake’ state (Listing 3, L18: all pixels flash white at maximum luminance for 100 ms, and then remain off, 〈0,0,〉).

Next a ‘handshake’ is performed between Matlab and Arduino. A handshake is an automated process that occurs prior to the transmission of content, and is used to set parameters and ensure that both systems are in a suitable state to proceed. In the simple example shown here, the handshake ensures that neither the Matlab or Arduino scripts continues until each has sent and received a predetermined passcode (Listing 1, L18–29; Listing 3, L81–107). If the Matlab script does not receive a passcode within a certain period (default: 10 s) then the script will throw an error. This helps with debugging, and prevents the two scripts from becoming out of synch. In practice, the handshake is performed by having the Arduino continuously broadcast an arbitrary passcode character (in this case ‘Z’), and checking for an arbitrary input passcode in return (in this case ‘A’). In Matlab, the program waits to receive the Arduino passcode (‘Z’) before transmitting its own passcode (‘A’). At this point, the system is ready to be used.

Once the ‘setup’ function is complete, the Arduino will automatically execute the ‘loop’ function (Listing 3; L39–79), and will continue to do so indefinitely, so long as the board is supplied with power. In Listing 3, the loop function causes the Arduino to continuously check the serial port for incoming control commands, which are then executed. These control commands are broadcast from Matlab using the following, arbitrary communication protocol:

1 $$ CN\backslash n $$

where C is the character ‘*’ (on) or ‘!’ (off), ‘ *N* ^′^ is an integer specifying the pixel number (where indices start at 0), and ‘ ∖n’ is a linebreak character, signifying the end of the command. Thus, the Matlab expression serialObj.println(‘*9’) will cause the 10 ^th^ LED Pixel to turn on, while the expression serialObj.println(‘!0’) will cause the 1^st^ LED Pixel to turn off (note that ‘println()’, unlike ‘print()’, automatically appends the ‘ ∖n’ line character to the end of the user-defined string). Alternatively, the user is free to implement whatever communication protocol they like. For example, it is possible to imagine a system in which multiple LED Pixels are updated at once within a single command, each with a different luminance, hue and temporal periodicity.

Within the Arduino microcontroller, command strings are received as individual characters, sent in series over the USB 2.0 connection (e.g., Listing 1; L41, L45). These characters are accumulated in a buffer within the Arduino until a linebreak character (‘ ∖n’) is reached (Listing 3; L49). At this point the command is evaluated and executed, and the buffer is cleared (Listing 3; L50–70). The actual interface with the LED Pixel hardware is mediated by the (freely available) Adafruit_WS2801 Arduino library. Note that the logic of this library is that separate commands are used to update an internal table of LED luminance values, and to display the update values. Thus, it is possible to send multiple update commands, before triggering the display change (i.e., just as with a computer monitor, multiple commands may be written to an offscreen buffer at times *t*_1_ and *t*_2_, before flipping this buffer onto the display at time *t*_3_).

As well as receiving data over the serial port, the Arduino is also able to write information back to the host computer, using Serial.print() commands. Thus, after executing a command, the code in Listing 3 (L72–76) transmits a message confirming that the command was completed. If, as in Listing 1 (L31–34), a Matlab callback function was specified, then this will automatically execute on receipt of any incoming data. In Listing 2, the logic of this code is the same as in the Arduino Loop() function. Namely, incoming serial data is accumulated in a buffer until a linebreak character is received, at which point the information is processed (in this case by simply printing the message to the Matlab console; Listing 2, L1–33). Note that these callback commands are executed asynchronously from the main control script. This means that although the callback code in Listing 2 is executed in the same thread as the main script (Listing 1), it can be executed at any time (i.e., calling Listing 2 briefly pauses the main Matlab script, which then automatically resumes upon completion). This means that the main script does not have to wait to receive the information before proceeding (e.g., to the next trial). However, in some circumstances it may be beneficial to force the program to pause to await incoming data, for example if the Arduino is expected to return important information that must be saved at the end of each experimental trial. Note also that although in the present example the data returned from the Arduino is simply printed to the Matlab console, it could equally be saved to a hard disk, or used to directly control the behavior of the main Matlab script.

Once the main Matlab experiment script is complete, it closes the serial connection with the Arduino, and releases any demands on memory (Listing 1; L48–51). Note that disconnecting the serial connection will cause the Arduino board to reset and the ”Setup()” function to be executed (Listing 3, L13–37), just as opening the serial connection did in step one. In practice, this will cause the LED Pixel library to be reinitialized, and all the LED Pixels will be turned off. Because of the use of a handshake step, the Arduino script should then remain in the ‘awaiting handshake’ phase (Listing 3, L84–107) indefinitely, until a new serial connection is establish and the Arduino script once again resets.

## Characterization

To characterize the properties of the Adafruit 12mm diffused LED Pixels (see section “Hardware: Overview”), measurements of luminance color spectrum, and temporal response were made (Leachtenauer 2004; Brainard et al. 2002) Details of how recordings were made are given within the relevant subsection. Where stated, analogous recordings from an example Cathode Ray Tube [CRT] monitor (ViewSonic G90fB 19”; ViewSonic Corporation, Brea, CA, United States) and liquid-crystal display [LCD] monitor (Samsung SyncMaster 305T 30”; Samsung Electronics Co., Ltd., Seoul, South Korea) were also taken for comparison. Further details of those recordings are reported in the Supplemental Material Statistical tests were evaluated at the 95 % significance level (*α*= 0.05).

### Luminance measures

Luminancemeasurementsweremadeusinga Minolta CS-100colorimeter (Minolta Camera Co., Osaka, Japan) fitted with a 1.3mm zoom lens (Minolta Model No. 122)

#### Input/Output (gamma) function

**Fig. 4:**
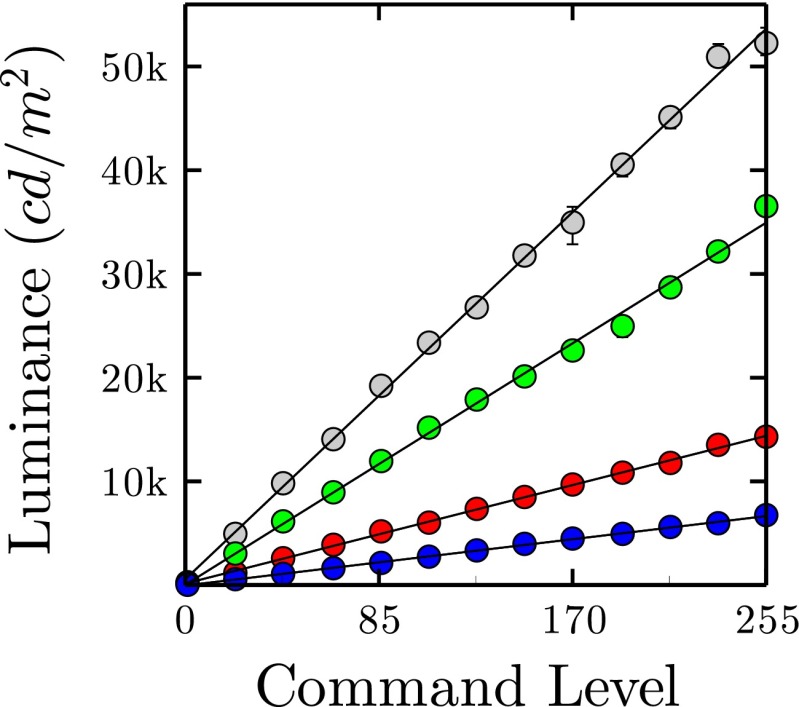
Gamma function: mean (± 1 S.E., across five LED Pixels) luminance output, as a function of command level input. Shown for a white light, and for each red, green, blue LED presented in isolation. *Error bars* not visible when standard error < marker size

To characterize LED gamma response, luminance measurements were made as a function of command [input] level. Measurements were made at 13 uniformly spaced command levels (CL = 0–255, inclusive). Measurements were made for a white light (same CL values in each RGB channel; Fig. 4a black line), and also for each color channel in isolation (Fig. 4a colored lines). This procedure was carried out independently for ten uniformly spaced LED Pixels (one set of measurements per LED Pixel).

As shown in Fig. 4, the luminance profile of the LEDs was highly linear (all r^2^> 0.98), and each LED Pixel was capable of producing a wide range of luminance levels (white: 250–52,250 cd/m^2^). There was also very little variability between LED Pixels, indicating that their response is highly stable (e.g., across input levels, the mean coefficient of variation between white-light LED Pixels was 4.9 %). To put these values in context, the luminance of the LCD screen varied by 15.0 % across its spatial extent.

From the Input/Output function, it is also possible to derive a number of other useful measures (Leachtenauer 2004), such as Dynamic Range (23.2 dB), Dark Cutoff (1), and Gamma (2.40).

#### Maximum acceptable viewing angle

**Fig. 5:**
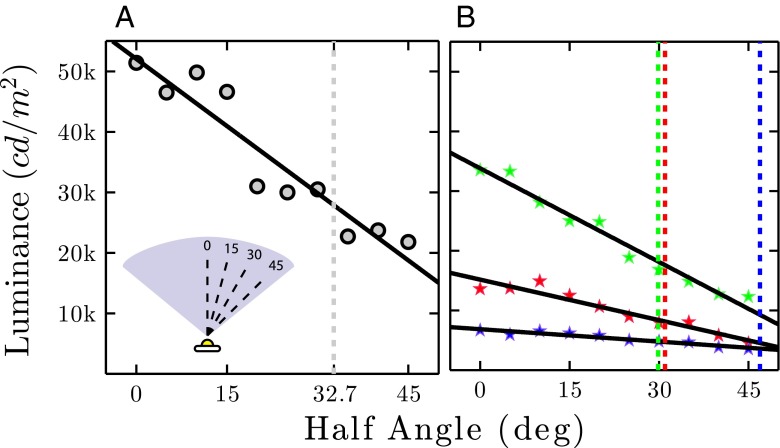
**a** Half-luminance viewing angle (HLVA) for a full-intensity white light. The *vertical dashed line* indicates the largest angle at which measured luminance was 50 % of the maximum. Variability in luminance not accounted for by the least-square linear fit (r ^2^= 0.89) is likely due to human error in where the colorimeter was positioned relative to the center of the LED Pixel (i.e., error in the *x*-axis of the graph). See Supplemental Material (Fig. S1) for analogous recordings from a CRT and LCD monitor. **b** Analogous recordings for each of the three individual RGB elements

The luminance of an LED tends to be highly dependent on the angle at which it is viewed. To quantify this directionality, maximum acceptable viewing angle was operationalized as half luminance viewing angle (HLVA)—the largest angle at which the measured luminance of a full intensity white light was 50 % of the maximum (as measured at 0 ^∘^). The result, shown in Fig. 5a, was HLVA =± 32.7 ^∘^, corresponding to a full (left-to-right) span diameter of 65.4 ^∘^. This range of viewing angle is approximately half that of the LCD screen (HLVA _LCD_ = 62.4 ^∘^) and smaller still than that of the CRT screen (HLVA _CRT_= 82.5 ^∘^)—CRT technology being highly tolerant of off-angle viewing (Krupinski et al. 2005). From these results, it can be concluded that a potential limitation of LED Pixels are their directionality, making them best suited to situations where the stimuli are never viewed obliquely (e.g., as in Fig. 1b), and/or where variations in stimulus intensity are not a concern. The luminance measurements for each individual RGB element (Fig. 5b) were qualitatively similar to those for a white light (R: ± 32.7^∘^ ; G: ± 29.8^∘^ , B: ± 46.9 ^∘^), and indicated a linear dependency of luminance on viewing angle. Note that the different slopes for the three color channels means that precise hue of any additively mixed colors is liable to vary with viewing angle, though subjectively these variations are not typically salient (see section “Characterization of chromaticity” for data regarding chromaticity).

#### Drain and halation

Drain describes a bleeding effect, whereby the luminance of one LED Pixel is reduced when those around it are turned on also (i.e., due to a decrease in available current). Drain is a conspicuous problem for most output displays, including LCD and CRT monitors, where dependencies between neighboring regions of the screen can result in stimulus artifacts of sufficient magnitude to confound a precise psychophysical experiment (Garcia-Perez and Peli 2001).

**Fig. 6:**
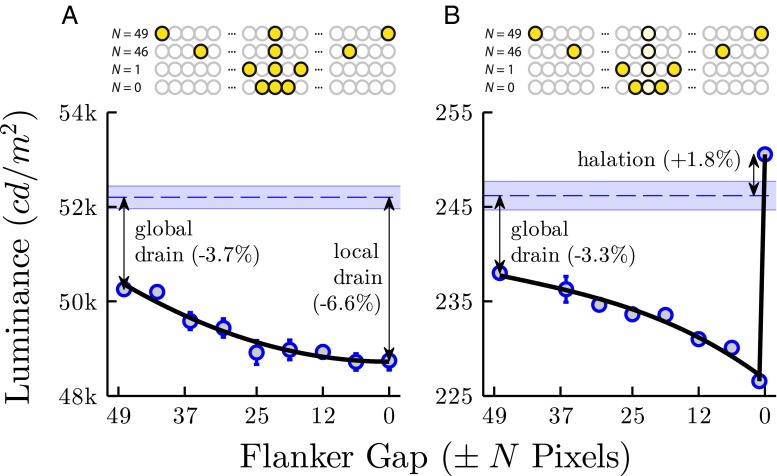
Drain **a** and halation **b**. Markers show mean (± 1 S.E.) luminance for a single LED Pixel (same throughout), as two flanker pixels are brought progressively closer to the target. See body text for details. See Supplemental Material (Fig. S2) for analogous measurements for each individual RGB element

Drain was assessed by measuring output luminance at a fixed, central LED Pixel (the target), as LED Pixels at two other locations (the flankers) were illuminated with increasing proximity (as illustrated in Fig. 6). Both target and flankers were set to maximum luminance throughout (command level = 〈255, 255, 255〉). Drain was quantified as percentage difference to mean target luminance when no flankers were present (Fig. 6a, horizontal dashed line). The result is shown in Fig. 6a. Drain effects were small but clear, and increased progressively with proximity. When the flankers were relatively distal (global drain), target luminance was reduced by 3.7 %. When the flankers were immediately adjacent to the target (local drain), target luminance was reduced by 6.6 %. (N.B. flankers were physically occluded from the colorimeter to prevent measurement confounds).

Halation is the inverse of drain, whereby a dark (low luminance) pixel surrounded by bright (high luminance) pixels, exhibits an increase in luminance (i.e., a higher luminance response than would be predicted by the input command level alone (Leachtenauer 2004)).

As indicated in Fig. 6b, halation was assessed by repeating the drain measurement procedure, but setting the target LED Pixel output to its minimum measurable level (command level =〈1, 1, 1〉^2^). Global drain was largely unchanged, (compared to the previous test using a maximum level target), but the effects of local drain were swamped by halation, which resulted in luminance *increasing* by 1.8 % (compared to the 6.6 % decrease due to local drain, measured previously).

To put these data in context, the just noticeable difference [JND] for detecting a change in luminance is approximately 2 % for a white light presented against a dim-photopic background (3–40 cd/m ^2^), rising to around 3 % for a 100 cd/m ^2^ background (comfortable viewing level of a monitor; see Supplemental Material for more info on typical luminance-detection JND values). The effects of halation reported here will therefore not be generally apparent to observers, and so will be of little concern for many behavioral scientists. However, the effects of drain may be detectable under close inspection. If performing stringent psychophysical procedures (e.g., where perception of luminance is the measure of interest), it would therefore be advisable to correct for the variations in luminance caused by drain, particularly when presenting ‘flanking’ stimuli immediately either side of the target.

**Fig. 7:**
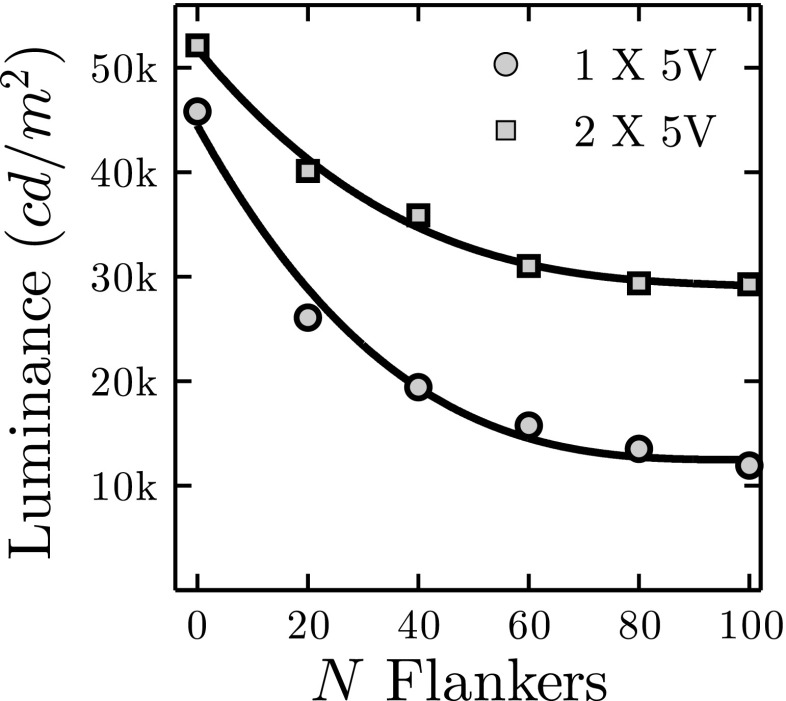
Drain effects for increasing dense displays. Each point gives mean (± 1 S.E.) luminance for 12 measurements of the same, ‘target’ LED Pixel. *Error bars* were smaller than marker size in all cases and so are not visible. The target was the central LED Pixel in a strand of 123 LED Pixels. The flanker gap was fixed at ± 10 pixels, and the number of flankers was varied, with half either side of the target. The target and any flankers were set to maximum luminance (RGB =〈255,255,255〉)

Moreover, it is important to note that the effects of drain can be exacerbated by increasing the number of flankers. For example, when the number of flankers was increased to 100 (with a ±10 LED notch around the target), target luminance decreased by 74 % (Fig. 7, circles). As shown in Fig. 7 (squares), such drain was mitigated but not eradicated (74 % => 44 %) by connecting a second power supply to the trailing end of the strand. It is likely that connecting additional power inputs would have further reduced drain, but this was not tested. For dense LED Pixel displays requiring precise absolute luminance levels, careful calibration, and multiple power supplies, may therefore be required.

### Spectral measures

Spectral measurements were made using a telescopic spectroradiometer (Gamma Scientific, San Diego, CA, USA). Measurements of overall color gamut were also made using the CS-100 colorimeter detailed previously (Section “Luminance measures”).

#### Power spectral density [PSD]

**Fig. 8:**
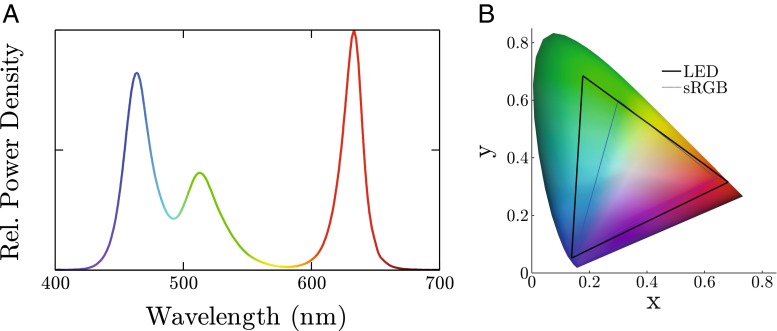
**a** Relative power spectral density of a LED Pixel at maximum luminance (i.e., relative whitelight irradiance as a function of wavelength). **b** CIE (1936) color space chromaticity diagram, with *triangles* showing the gamut of the LED Pixels (*thick black*) and the sRGB industry display standard (*thin blue*)

The power spectral density (PSD) of the LED Pixel, measured for a white light at full brightness, is shown in Fig. 8a. Unlike a laser (sinusoidal spectra) or an incandescent bulb (broadband spectra), an LED has a narrowband spectral response. The individual response from each of the three constituent LEDs is therefore clearly visible in Fig. 8a. The peak output wavelengths of the three elements were 464.5, 512.3, and 633.4 nm, and they approximately followed normal distributions with standard deviations of 16.7, 25.5, and 12.2 nm^3^ (respectively). This corresponds to full width half modulation [FWHM] values of 39.3 (blue), 60.0 (green), and 28.7 nm (red). These response spectra mean, for example, that the ‘red’ LED element emits extremely little light below 600 nm. Since rod photoreceptors are highly insensitive to wavelengths above 600 nm, the ‘red’ LED element can therefore be particularly useful for isolating cone functionality; for example, in order to monitor residual function in cone dystrophies such as achromatopsia (Moore 1992). The width of the spectral power distributions for each of the three color channels were similar to those reported previously for CRT phosphers (Brainard 1989), though the ‘blue’ and ‘green’ elements were somewhat more narrowly distributed.

#### Color gamut

Color gamut describes the subset of visible hues that can be produced reliably by the device. The industry, sRGB, standard for commercial display devices (Anderson et al. 1996) is shown by the thin blue line in Fig. 8b. Compared to this, the LEDs perform favorably (Fig. 8b, thick black line). They were able to produce 99.9 % of the sRGB colorspace, along with substantially deeper hues of green.

#### Characterization of chromaticity

To further characterize the chromatic properties of the LED Pixels, many of the previous analyses of luminance were repeated using chromaticity instead of luminance as the dependent variable. Chromaticity was defined in terms of the u’ and v’ chromaticity coordinates of the CIE 1976 color space^4^ (a.k.a. CIELUV) (Robertson 1977). Values of 〈L, u ^′^, v ^′^〉 were computed from the recorded x/y coordinates using the OptProp Matlab toolbox (Wagberg 2007), assuming a D65 illuminant and a 2 degrees observer.

A difference in chromaticity (“Chromatic Shift”) was defined as the Euclidean distance of the observed 〈*u* ^′^, v ^′^〉 coordinates from the ideal. The ideal coordinates, $\langle \textit {u}^{\prime }_{\text {ideal}}$, v$^{\prime }_{\text {ideal}}\rangle $ were defined as those measured when the same pixel was: (i) set to maximum luminance, 〈255,255,255〉; (ii) measured from straight on, 0 ^∘^; (iii) switched on for 10 min to allow for any potential warm-up (though warm-up effects are shown below to be negligible). Thus, lower values of chromatic shift indicate greater stability in terms of hue and saturation. Although there is no universally accepted JND value for chromaticity, here we took 0.003 (a three-step MacAdam Ellipse (Wyszecki and Stiles 2000; Royer et al. 2013; Mahy et al. 1994)) as the minimum unit of discriminability when considering whether observed chromatic shift values were substantive.

**Fig. 9:**
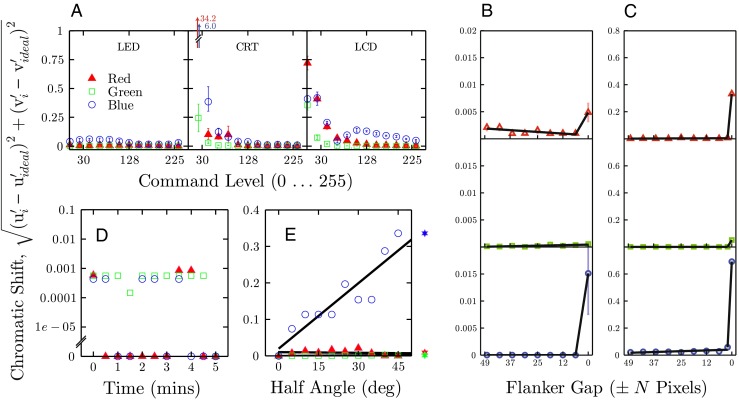
Changes in LED Pixel chromaticity coordinates (± 1 S.E.) as a function of **a** input command level, **b** drain, **c** halation, **d** warm-up, and **e** viewing angle. The out-of-axes markers in (e) indicate additional measurements made at + 70 ^∘^. The numeric values for (a) are given in the Supplemental Material (Table SI)

From inspection of Fig. 9, it can be seen that the LED Pixels generally exhibited good chromatic stability. Compared with CRT and LCD technology, chromaticity was much less dependent of luminance (Fig. 9a). The green element in particular was highly consistent, exhibiting almost constant chromaticity across all luminance levels (≤ 0.001; see Supplemental Material for details). The red element was also relatively stable across luminance levels, exhibiting chromatic shifts on the order of ∼ 0.006. This would be detectable, though only under close inspection. The blue element was substantively less stable (∼ 0.036), exhibiting luminance-dependent variations in chromaticity which would need to be corrected for if performing precise psychophysical procedures. However, these variations in chromaticity were still markedly smaller than those exhibited by CRT (∼ 0.560) or LCD (∼ 0.153) devices^5^, and would not be noticeable under casual viewing. In the LED Pixels, the smallest programmable increment in chromaticity (e.g., 〈128,128,128〉 vs. 〈128,129,128〉) produced a mean chromatic shift of 0.012.

Effects of drain (Fig. 9b) and halation (Fig. 9x) were generally either zero or negligibly small (< 1 JND). One notable exception to this overall trend was halation caused by immediately adjacent LED Pixels. Thus, when a maximally intense white light was presented next to a dim red, green, or blue light, there was some visible distortion in the color of dim light. Such effects could be avoided in practice by leaving a one pixel ‘buffer’ around very dim lights. Warm-up times were negligible, with no systematic variations in chromaticity over time (Fig. 9d), confirming that a warm-up period is not required when using LED Pixels. Finally, chromaticity was highly stable with viewing angle for the red and green elements, but was substantive confound for the blue element. Thus, as followed previously from considerations of luminance (section “Maximum acceptable viewing angle”), it would be important to always view the LED Pixels at a constant (e.g., perpendicular) angle if precise stimulus constancy were required.

### Temporal measures

Timing measurements were made using a CRS LM03 photodiode (Cambridge Research Systems, Cambridge, UK), sampling at a rate of 5 *μ*s.

#### Response time and onset lag

**Fig. 10:**
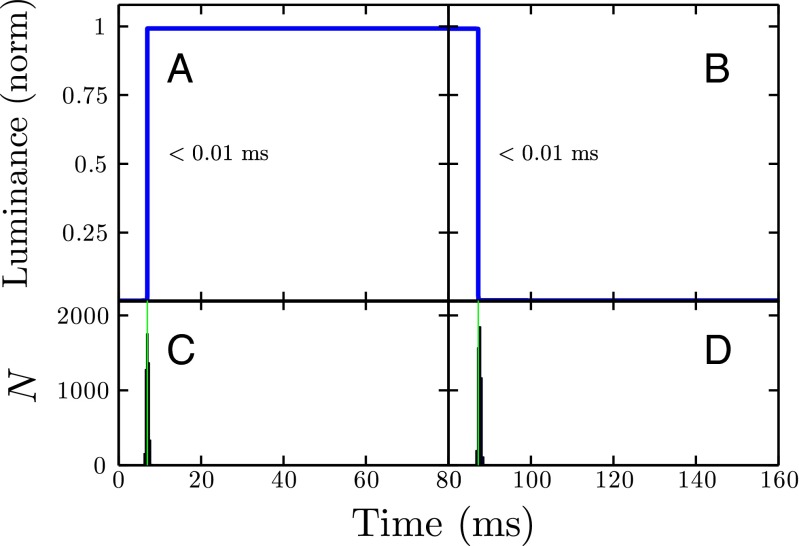
**a**, **b** Response time curves, averaged over 10,000 off-on (**a**) or on-off (**b**) transition. Individual measurements were aligned temporally via crosscorrelation prior to averaging. Panels **c** and **d** show the distribution of cross-correlation lag times (i.e., amount of trial-by-trial lateral variability in the response curve shown above). The shape of the curves in the upper panels indicates the response time. The distributions in the lower panels indicate mean onset lag (*green vertical line*), and variability in onset lag (*histograms*)

Response time was measured as the number of milliseconds taken for the display device to transition from fully off to fully on (black-to-white response time; BWRT), or from full on to full off (white-to-black response time; WBRT). As can be seen in Fig. 10a, b, the results from the LED Pixels were indistinguishable from a step function, meaning that the response time was virtually instantaneous (< 0.001 ms). This compares favorably with the LCD display (BWRT: 7.46 ms; WBRT: 6.68 ms), and is faster even than the impulse response time of the CRT (BWRT: 0.58 ms; WBRT: 2.45 ms), details for which are given in the Supplemental Material (see also Elze and Tanner (2012) for a more detailed and comprehensive analysis of LCD technology)

A related measure to response time is onset lag: the duration between a command being sent and it being actualized by the display. This includes both the response time and any other limitations due to transmission speed and refresh rate. From Fig. 10c it can be seen that onset lag was ∼ 6 ms. This compares favorably with the typical refresh rates of an LCD or CRT monitor (60–120 Hz; ∼ 8–16 ms), and means that the LED Pixels can be manipulated in near real time, and with a far higher temporal fidelity that can be achieved using a standard LCD or CRT monitor. The LED Pixels are therefore well suited to situations where precise control of temporal duration is required—for example to maximize responsiveness when designing an interactive (user controlled) display.

There was also very little variability in onset lag across repeated observations (Fig. 10c, d, vs. Figs. S3, S4 in the Supplemental Material). This means that the temporal response of the LED Pixels is not only fast but also highly reliable/predictable. Such fast and reliable response times are especially appealing to users looking to synchronize the visual output with a secondary output, such as an audio device.

#### Refresh rate

**Fig. 11:**
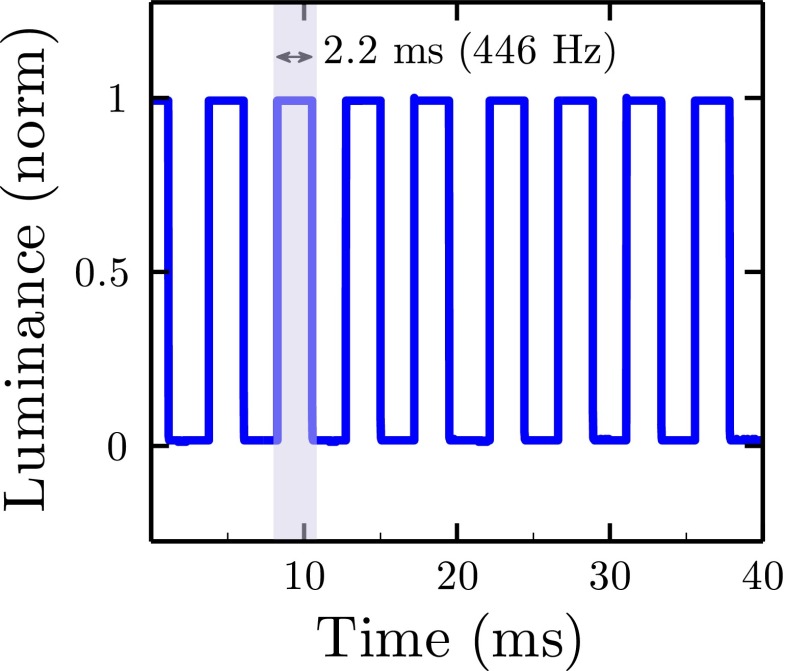
LED Pixel refresh rates. Curve shows relative luminance output (normalized by dividing by maximum observed level) as a function of time, as a single LED Pixel was turned on/off without any user-specified delay. *Highlighting* shows a single, example sustained duration, which lasted 2.2 ms

An onset lag of 6 ms (see section “Response time and onset lag”) corresponds to a potential refresh rate of 167 Hz. This is already faster than most commercial monitors, which typically operate at 60–120 Hz. Notably though, the 6-ms lag is due in part to the time taken to encode/transmit/decode commands sent in series over the USB port. Thus, even higher refresh rates can be achieved by sending whole sequences of commands to the microcontroller in advance (e.g., to be executed at specified time in the future, or following a predetermined trigger). For example, Fig. 11 shows an LED Pixel following a predetermined on/off sequence. Here, a refresh rate of 446 Hz was possible. Thus, LED Pixels may also be particularly well suited for experiments that require a high flicker rate (Brindley et al. 1966; Simonson & Brozek 1952).

Note, however, that a refresh rate of 446 Hz is an upper limit, and the maximum refresh rate decreases as the number of LED Pixels increases (N.B. where *N* is the number of LED Pixels specified when the WS2801 Arduino library is initialized—Listing 3, L9—and *not* how many LED Pixels are physically connected). This reduction in refresh rate is because, while it takes the same amount of time to ‘flip’ one pixel as it does for an entire strip (the ‘show’ command; Listing 3, L70), luminance update commands are briefly ‘held’ by each LED Pixel, and so incur a cumulative time cost (the ‘setPixelColor’ command; Listing 3, L69). As a consequence, it can take longer than 2 ms (446 Hz) to update the luminance levels of even a single LED Pixel if a large number of LED Pixels have been specified as addressable. For applications requiring exceptionally high refresh rates (e.g., 446 Hz), therefore only one or two LED Pixels can be addressed

#### Warm-up rate

In section “Response time and onset lag” we discussed response time: how long it takes for the device to transition from one state (e.g., minimum luminance) to another (e.g., maximum luminance). With traditional visual output devices such as CRT and LCD monitors, there is also a longer-term dynamic in which maximum luminance increases gradually for a period after the device is turned on. Thus, the maximum luminance output of an LCD or CRT screen will be greater after 60 min than it is when first powered up for the day, though most of this change typically occurs within the first 5–10 min (Bird 2010). With LED technology, such *warm-up* effects are still a potential concern, since the temperature at the junction of the LED chip can affect forward voltage, and thus the amount of light emitted.

**Fig. 12:**
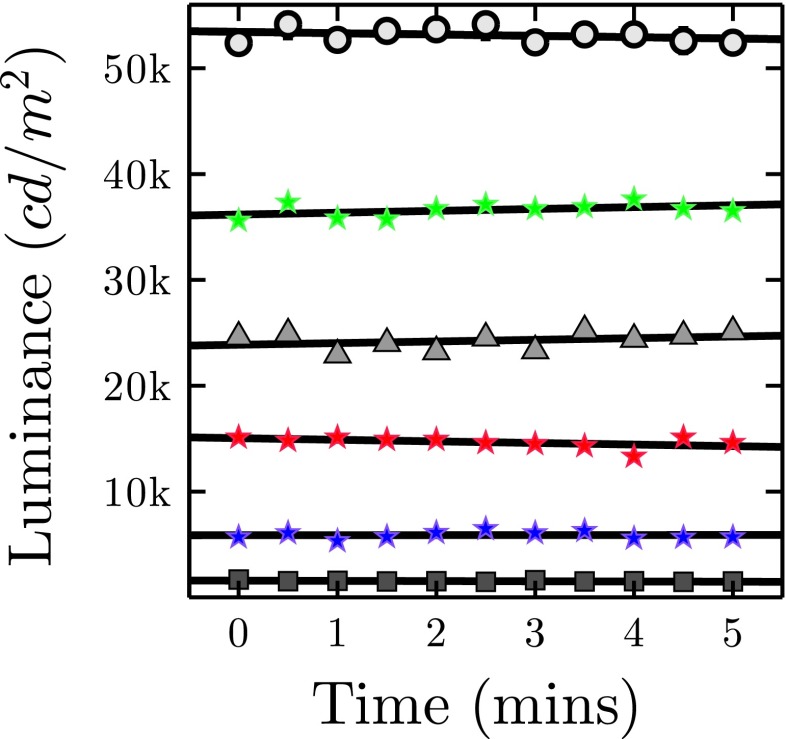
LED Pixel warm-up dynamics, showing luminance measurements for three grey levels (CL = 8, 128, 255), and for each of the three RGB color elements (CL = 255), as a function of time. *Markers* show mean (+ -SE) luminance levels for each of seven LED Pixels, measured independently (*error bars* not visible when smaller than marker). *Lines* represent least-square regression fits, and did not differ from zero in any case (no change in luminance with time; see body text for details)

To examine whether LED warm-up is a practical concern for behavioral scientists, measurements were made every 30 s for LED Pixels that had not been powered in the previous 24 h. The results are shown in Fig. 12, and indicated that no warm-up effect was detectable at low (*t* test comparison of linear regression slope to zero: *t*_9_= -2.01, *p*=.076, *n*.*s*.), medium (*t*_9_= 1.05, *p*=.323, *n*.*s*.), or maximum (*t*_9_= -0.94, *p*= 0.372, *n*.*s*.) luminance settings. Thus, while we cannot rule out extremely small, extremely rapid, or extremely gradual effects, warm-up is unlikely to be a concern when using LED Pixels. This makes them more convenient than traditional visual displays (which typically need to be turned on at least 30 min prior to testing when a high degree of precision is required), and eliminates a potential source of measurement error.

## Discussion

The present paper describes a simple, cheap, and flexible solution for controlling LED light sources, using digitally addressable LED Pixels connected to an Arduino microcontroller. It also details the properties of such a system, in terms of output luminance, color, and temporal precision.

In some instances, such a system can provide unique advantages over traditional LCD or CRT monitors. For example, LED Pixels were shown to have exceptionally fast and reliable response properties, narrowband color spectra, and a wide dynamic range of luminance levels. Each of these properties may make them appealing for psychophysicists, as might their high degree of linearity (which makes them extremely easy to calibrate and manipulate). For more general users, their ease of use and flexibility may make LED Pixels an attractive proposition in a range of settings. For example, the fact that LED Pixels can be placed anywhere, either in isolation or in clusters, makes them especially well suited to more ‘ecologically valid’ experiments (i.e., where stimuli are presented in a real-world environment, rather than on a screen or headset). As noted by previous authors (Teikari & et al. 2012), LED technology may also be particularly suited to experiments employing electrophysiological equipment, due to their relatively low electromagnetic interference emissions.

The potential caveats of the LED Pixels were shown to be their relatively narrow viewing angle (i.e., appearing substantially less bright when viewed eccentrically), and the fact that both timing and luminance properties were dependent on the number of LED Pixels used (i.e., the greatest luminance and refresh rates were only possible when using a single LED Pixel). It should also be noted that the LED Pixels presented here do not support analog (direct current) dimming, though their relatively high PWM means that this will not be a limiting factor for the majority of users (see the Supplemental Material for discussion).

The hardware described here is easily extensible. The number of lights can be increased simply by clipping together additional LED Pixels. More generally, the basic computer/microcontroller setup can also be adapted for qualitatively distinct purposes. For example, previous authors have detailed more complicated setups in which an Arduino microcontroller is combined with custom-built circuit boards to control both LED elements and incandescent (civil aviation standard) light bulbs, in order to model the effects of different light sources on visual perception and action (Gildea & Milburn 2013). Furthermore, the same basic Arduino system (D’Ausilio 2012) can be easily extended to send and receive data from other devices, including both other forms of outputs (e.g., motors, servos, piezoelectric speakers), as well as various forms of input sensors (e.g., temperature, geolocation, galvanic skin response, compass direction).

Finally, it is worth stressing that the system described here only requires the plugging together of standardized, off-the-shelf components. This makes it a cheap and easy-to-use solution for users with a wide range of technical expertise, and the large amount of online support means that users are likely to be able to find help if/when problems occur.

## Electronic supplementary material

Below is the link to the electronic supplementary material.

(PDF 364 KB)

## Footnotes

1 In fact, modern LCD monitors exhibit marked heterogeneity. Different flat-screen technologies exhibit distinctive response characteristics, and response characteristics may even differ between screens with identical model numbers, either randomly, or as hardware/firmware is changed or updated. Moreover, even within an individual screen, response behavior is often highly dependent on where on the screen the stimulus is presented, and on what is being displayed contiguously with the stimulus (either in time or space). Finally, the behavior of LCD monitors is very differently to that of older CRT monitors, which themselves are now often highly variable due to aging components and their own inherent non-stationarities.

2 N.B. Unlike LCD screens, an input of 〈0,0,〉 produces no measurable luminance output, and so could not be used to assess the effects of Global Drain.

3 Note that the optical bandwidth of a spectroradiometer is liable to broaden the spectral shape of a measured source. The true values may be marginally lower.

4 The CIELUV color space is designed to be perceptually uniform, such that two colors that are equally distant in the color space are equally distant perceptually.

5 NB recordings were made in dark (mesopic) conditions, so ambient/external flare would be minimal. However, in the case of the LCD device, chromaticity at low command levels will have been confounded by internal flare due to backlighting (Fernandez-Maloigne 2012). Such a chromatic shift can be straightforwardly corrected for. However, even at higher command levels (> 100), the mean level of chromatic shift in the LCD screen, 0.094, was approximately four times greater than that observed in LED Pixels (0.024)
