# Supplementary material for: Digital LED Pixels: Instructions for use and a characterization of their properties
Source: Behav Res Methods. 2015 Oct 20;48(4):1266–84. doi: 10.3758/s13428-015-0653-5 (PMC5101347; doi:10.3758/s13428-015-0653-5)
Supplement: Supplementary file 1 — (PDF 364 KB) [file 13428_2015_653_MOESM1_ESM.pdf]

Supplemental Material
Digital LED Pixels: Instructions for use and a characterization of their properties

Pete R. Jones, Sara Garcia, and Marko Nardini

1. A note on direct current luminance modulation

One way to vary the luminance of a light source is by varying its input voltage (and thereby the amount of current flowing through the device). This can be done either by inserting a potentiometer — 'variable resistor' — between the power source and the device, or by using a digital to analog converter [DAC] to vary the dc component of the analog input signal. The primary shortcoming of direct current luminance modulation is that it can also cause hue to co-vary with intensity (e.g., see Figure 2 of Ref [9] in main text).

Instead, the LED Pixels reported here use Pulse Width Modulation [PWM] to control luminance. Under PWM, an LED element is always fully off (0%) or fully on (100%), but can alternate rapidly between these states. Integrating over time, average luminance can therefore be controlled by varying the proportion of time that the LED is on for. Since the instantaneous forward current is always constant, hue does not vary with luminance as it does with analog dimming. PWM therefore provides precise control of luminance, independent of hue (see §4.2.3 in main text). Moreover, since the alternations occur very rapidly, this flicker is not generally apparent to observers. For example, the reported LED Pixels have a PWM rate of 2500 Hz, compared to a foveal Critical Flicker Fusion Threshold of around 50 âĂŞ 60 Hz in humans31,32.

However, in certain situations the use of PWM may confound very precise psychophysical measurements. For example, flashing due to PWM may become apparent when a dimmed LED is moved rapidly across the retina33. Unfortunately, although LED drivers that support direct current modulation are available, a drawback of the reported LED Pixels is that they only support dimming via PWM. Moreover, given the confounds between hue and luminance, digitally addressable LED Pixels that support analog dimming are likely to remain of limited commercial appeal.

For users looking to minimize temporal modulation effects, the simplest solution would be to substitute the LED Pixels reported here, with those that support a very high PWM rate. For example, the latest 'Adafruit DotStar' pixels (Adafruit Industries, New York, USA) support a 20,000 Hz PWM rate, which ought to be psychophysically indistinguishable from a continuous signal, even during eye/stimulus movements. Alternatively, for users who absolutely require a continuous light source of variable luminance, various products exist that do support analog dimming of individual LED elements via an Arduino microcontroller. For example: (i) the Power LED Arduino shield (www.chestersgarage.com) uses potentiometers to drive four independent LED channels that can be controlled 10-bit fidelity; (ii) the latest Arduino Due microcontrollers (SmartProjects, Strambino, Italy) have two integrated DAC channels which allow voltage to be varied between 0.55 and 2.75 V, with 12-bit fidelity; (iii) a number of manufacturers provide external DAC breakout boards that provide 12- to 16-bit fidelity, and can be stacked to provide multiple output channels. Notably though, all of these solutions would require users to manually wire individual LED elements to each output channel, and could not be used in combination with the digitally addressable LED

Pixels described in the present manuscript (i.e., all of which share a common input current). They would therefore not provide the convenience or chromatic stability of digitally addressable LED Pixels.

2. Additional measurement data

Here we present additional characterizations of an example CRT and LCD monitor. Shown here are data concerning: viewing angle (**Fig S1**), drain and halation (**Fig S2**), and temporal response times (**Fig S3**, **Fig S4**). **Table SI** also gives the raw numeric chromatic shift values for LED Pixel of varying input levels, as shown graphically in Figure 9A of the main text.

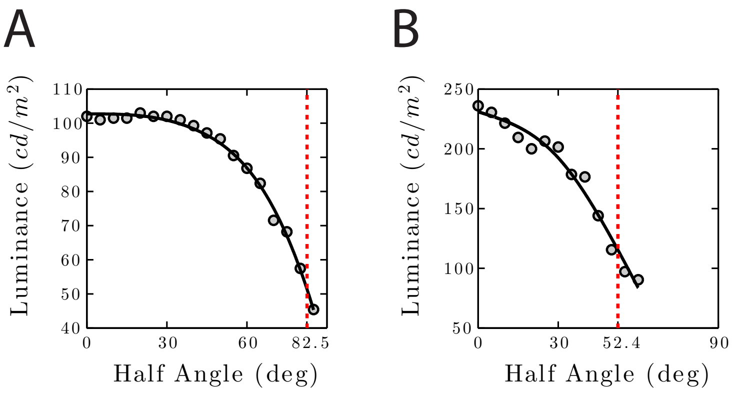

Fig S1. Minimal acceptable viewing angle recordings for a (A) CRT and (B) LCD monitor, in the same format as Figure 5 (Main Text). It should be noted that new IPS LCD panels, and next-generation OLED panels have much better viewing angles than the values for the Samsung 305T TFT display shown here.

Chromatic Shift: $\sqrt{(u'_i - u'_{255})^2 + (v'_i - v'_{255})^2}$

CL	LED			CRT			LCD		
	R	G	B	R	G	B	R	G	B
234	0.002	0.000	0.030	0.002	0.001	0.009	0.003	0.001	0.049
213	0.002	0.000	0.016	0.005	0.001	0.011	0.004	0.001	0.062
192	0.005	0.000	0.016	0.006	0.001	0.014	0.007	0.001	0.082
170	0.007	0.001	0.016	0.002	0.001	0.010	0.008	0.001	0.091
149	0.007	0.001	0.016	0.007	0.002	0.020	0.008	0.001	0.108
128	0.009	0.001	0.030	0.004	0.002	0.038	0.016	0.002	0.129
107	0.008	0.001	0.042	0.012	0.003	0.041	0.028	0.001	0.139
86	0.007	0.001	0.057	0.099	0.006	0.079	0.047	0.003	0.096
65	0.009	0.001	0.057	0.081	0.007	0.123	0.069	0.005	0.044
43	0.006	0.001	0.060	0.101	0.029	0.385	0.169	0.019	0.205
22	0.006	0.001	0.055	34.165	0.244	5.984	0.410	0.074	0.419
1	0.012	0.001	0.040				0.720	0.356	0.410

Table SI. Chromatic shift values associated with Figure 9A (Main Text), measured independently for the red, green, and blue channels of each display, as a function of command level.

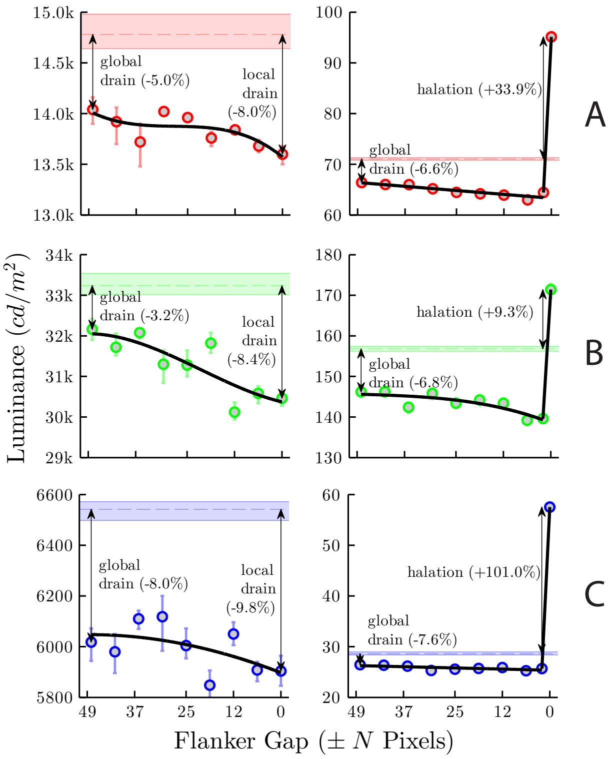

Fig S2. Drain and Halation measurements for individual (A) Red, (B) Green, and (C) Blue LED Pixel elements, presented in the same format as those for a white light in Figure 6 (Main Text).

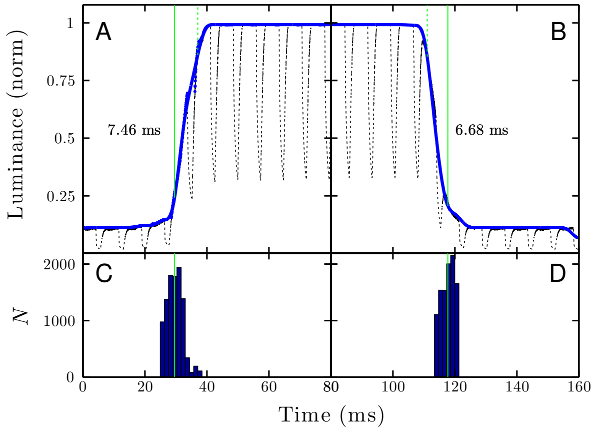

Fig S3. LCD response time measurements, for comparison with Figure 10 (Main Text). The dashed black line represents the raw data, which was fitted by a maximum curve (solid blue). The notches represent pulse width modulation [PWM], which is used in LCD screens to control luminance levels.

3. Just Noticeable Differences for luminance detection

Table SII shows the Just Noticeable Difference [JND] (i.e., the smallest percentage increment, required to distinguish a light from its adapting background), as reported in three classic visual psychophysics papers. Within the photopic range, the JND for de-

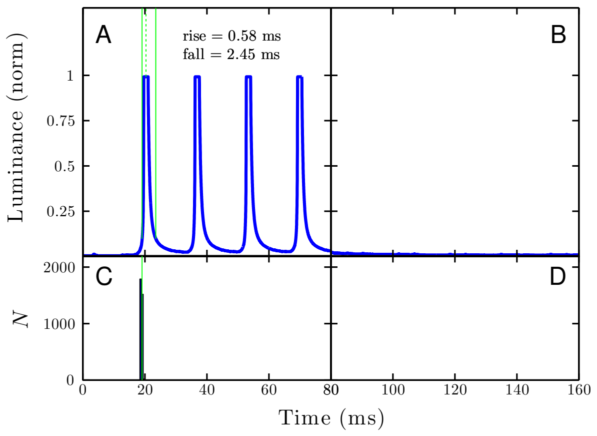

Fig S4. CRT response time measurements, for comparison with Figure 10 (Main Text). Note that no explicit offset-time curve was measured, as CRT monitors do not produce sustained, steady-state responses, but instead uses 'guns' to periodically fire electrons at the screen phosphors.

tecting a change in luminance is approximately 2% for a white light presented against a dim-photopic background (3 – 40 cd/m2), rising to around 3% for a 100 cd/m2 background. However, as shown by the data from Steinhardt (1936), sensitivity is decreased for smaller targets, and in general luminance detection thresholds vary depending on the luminance of the target, the spatial characteristics of the target, and the location on the retina (for an overview, see Blackwell, 1972).

In practice, observed thresholds are therefore often higher than the classically reported Weber fractions of 1 – 3%.For example, in the model of Ward and colleagues (Ward, 1994) the JND for luminance detection is approximately 14% when viewing a target presented against a 10 cd/m^2 adapting background, decreasing to approximately 6% for targets presented against higher luminance backgrounds. These values are broadly consistent with several empirical datasets (Brenton and Phelps, 1986; Maertens and Wichmann, 2013).

Luminance-detection JNDs ($100\Delta B/B$), as a function of background level (B)							
König & Brodhun (1889) 4.33°		Blanchard (1918) 5°		Steinhardt (1936) 4.67°		Steinhardt (1936) 0.5°	
B (cd/m^2)	JND (%)	B (cd/m^2)	JND (%)	B (cd/m^2)	JND (%)	B (cd/m^2)	JND (%)
116.50	3.5						
86.30	2.7						
57.85	2.6						
42.86	1.9						
31.75	1.7						
21.28	1.7						
15.77	1.7						
11.68	1.8						
7.83	1.8						
5.80	1.8			5.84	1.4	5.84	5.8
4.30	1.9			4.24	1.1	4.24	6.5
2.88	2.2	3.15	2.1	3.15	1.3	3.15	6.5
2.13	2.9	2.11	2.5	2.24	1.7	2.24	6.0
1.58	3.1			1.63	1.6	1.63	7.9
1.06	3.8	1.05	3.2	1.21	1.5	1.21	7.2
0.78	4.5	0.78	4.2	0.76	1.5	0.76	7.5
0.58	5.6	0.58	6.0	0.55	1.6	0.55	8.8
0.39	8.6			0.41	1.7	0.41	8.9
0.29	11.0	0.29	13.1	0.28	2.1	0.28	9.0
0.21	15.9			0.20	2.2	0.20	11.2
0.14	22.0	0.15	24.6	0.15	2.9	0.15	13.6
0.11	27.4	0.11	25.4	0.10	3.8	0.10	18.5
0.08	32.6	0.08	30.2	0.07	5.0	0.07	29.7
0.05	41.0	0.04	52.1	0.04	12.6	0.04	86.0

Table SII. Just Noticeable Differences [JND] in luminance, for a light presented against a background of specified luminance (B). The data from König & Brodhun (1889) and Blanchard (1918) were reported originally in units of millilumens, and were converted to candela/m^2 using the equation: $y_{cd/m^2} = \frac{x_{mL}}{0.1\pi}$. The data from Steinhardt (1936) were reported originally in units of photons, and were converted to candela/m^2 using the equation:$y_{cd/m^2} = \frac{x_{\gamma}}{10\pi r^2}$, where r is the radius of the exit pupil in millimeters (in Steinhardt, 1936, the radius of the exit pupil was fixed at 1mm, so the r term can be ignored). Note that data from König & Brodhun's 1889 experiments are taken from Blanchard, 1918).

Supplemental References

Blackwell, H. R. (1972). Luminance difference thresholds. In Jameson, D. and Hurvich, L. M., editors, *Handbook of Sensory Physiology: Psychophysics*, volume VII, pages 78–101. Springer.

Blanchard, J. (1918). The brightness sensibility of the retina. *Physical Review*, 11(2):81–99.

Brenton, R. and Phelps, C. (1986). The normal visual field on the humphrey field analyzer. *Ophthalmologica*, 193(1-2):56–74.

Maertens, M. and Wichmann, F. A. (2013). When luminance increment thresholds depend on apparent lightness. *Journal of Vision*, 13(6):21–21.

Steinhardt, J. (1936). Intensity discrimination in the human eye i: The relation of $\delta i/i$ to intensity. *The Journal of general physiology*, 20(2):185–209.

Ward, G. J. (1994). A contrast-based scalefactor for luminance display. In Heckbert, P. S., editor, *Graphic Gems*, volume IV, pages 415–421. AP Professional (Academic Press), Boston, US.
